# Habitat associations of Chihuahua Chub (*Gila nigrescens*) and Rio Grande Sucker (*Pantosteus plebeius*) in the Mimbres River, New Mexico

**DOI:** 10.1371/journal.pone.0341748

**Published:** 2026-02-19

**Authors:** Giulio W. Del Piccolo, Zachary B. Klein, Matthew P. Zeigler

**Affiliations:** 1 Department of Fish Wildlife and Conservation Ecology, New Mexico State University, Las Cruces, New Mexico; 2 New Mexico Department of Game and Fish, Fisheries Management Division, Santa Fe, New Mexico; University of Tehran, IRAN, ISLAMIC REPUBLIC OF

## Abstract

Fish species in arid regions have suffered from disproportionate declines due to anthropogenic activity. Therefore, many arid-region fishes are species of conservation concern and a primary focus of management agencies. Unfortunately, conservation of many of these species is stymied by a general lack of ecological information such as habitat use. In an effort to improve management of two species of conservation concern, we assessed the seasonal and life-stage specific habitat associations Chihuahua Chub *Gila nigrescens* and Rio Grande Sucker *Pantosteus plebeius* in the Mimbres River, New Mexico. Fish assemblage and habitat features were evaluated in the winter, spring, and summer of 2022 and 2023. Due to disparate data sets, habitat associations of Chihuahua Chub were analyzed using an *N*-mixture model and habitat associations of Rio Grande Sucker were assessed using linear regression. The relative abundance of Chihuahua Chub was associated with deep, structurally-complex pool habitats in reaches with dense riparian vegetation. Adult and subadult Rio Grande Sucker relative abundance was associated with low-velocity habitats. Subadult Rio Grande Sucker were associated with shallower habitat relative to adult conspecifics. Additional habitat restorations will likely benefit Chihuahua Chub and Rio Grande Sucker in the Mimbres River. This study informs the implementation of habitat restoration efforts to improve the conservation of both species across their distributions.

## Introduction

Fish species in arid regions of North America have suffered from a disproportionately high extinction rate over the last 100 years [1,2]. Habitat loss related to land-use changes and increased water demands is often cited as a primary contributor to these extinctions [3,4]. Conserving aquatic habitat is commonly used to maintain and/or restore sensitive species in arid regions [5,6]. Unfortunately, the effectiveness of habitat restoration can be limited by the incomplete understanding of a species’ habitat needs by season and life stage [7,8]. For example, emergency translocations and habitat maintenance for River Blackfish *Gadopsis marmoratus* in the Murray River basin, Australia, were hampered by a poor understanding of the habitat requirements of the species [9]. Conversely, increased stream flows and changes in riparian management were used to create deep, structurally complex habitats used by Modoc Sucker *Catostomus microps* in Turner and Ash Creeks, California which increased the abundance of the species [6]. Similarly, Arkansas Darter *Etheostoma cragini* habitat association were used to identify unoccupied stream segments containing suitable habitat for the species in the Arkansas River basin, Colorado, leading to the establishment of 10 naturally reproducing populations [10]. A similar understanding of habitat associations is needed to aid the conservation of many desert river fishes [11–13].

Chihuahua Chub *Gila nigrescens* and Rio Grande Sucker *Pantosteus plebeius* have both suffered from habitat loss throughout their distributions [14–16]. Chihuahua Chub occur in the Mimbres River, New Mexico, and the Guzman, Laguna Bustillos, and Rio Papigochic basins of Chihuahua, Mexico [17,18]. Rio Grande Sucker occur in the Mimbres River and upper Gila River basin of New Mexico, the Rio Grande basin of Colorado, New Mexico, and Chihuahua, the Guzman basin of Chihuahua, and the Rio Bavispe, Durango, Mexico [14,15]. Riparian habitat has been lost in much of the species’ distributions due to grazing, timber harvest, stream channelization, and decreased water availability [16,19,20]. Furthermore, recent wildfires (i.e., 2013 Silver Fire, 2022 Black Fire; [21,22]) and subsequent ash-flows have resulted in drastic reductions to the Chihuahua Chub population in the Mimbres River [16,23]. As such, Chihuahua Chub are considered “endangered” in New Mexico and Rio Grande Sucker are considered a species of conservation concern in the state [14,16]. Recovery efforts in the Mimbres River have included habitat improvements to increase the availability of deep and structurally complex habitats in the river as well as supplemental stocking of Chihuahua Chub [16].

Habitat improvements will likely increase the abundance and resilience to stochastic events (e.g., wildfire, dewatering events) of Chihuahua Chub and Rio Grande Sucker [14,23]. However, information on habitat association of both species is lacking. The habitat associations of Chihuahua Chub have never formally been quantified. Rio Grande Sucker habitat use has been evaluated, but this research largely occurred in the upper Rio Grande basin where the species is sympatric with nonnative White Sucker *Catostomus commersonii* [24]. The diets of White Sucker and Rio Grande Sucker both rely heavily on periphyton and Rio Grande Sucker have decreased body condition in sympatry with White Sucker [14,25]. Although this could confound habitat association information where the species co-occur, Rio Grande Sucker have been associated with low-gradient streams with cobble substrates [25,26]. Due to the limited information on the habitat associations of Chihuahua Chub and Rio Grande Sucker, the objective of our work was to assess the seasonal and stage-specific habitat use of both species.

## Methods

### Study area

The Mimbres River occurs in an endorheic basin in southern New Mexico [17]. Discharge in the river is typically low, but varies seasonally. Low flows typically occur in the late spring and early summer (mean June discharge = 0.15 m^3^/s; SE = 0.02; USGS gauging station number = 08477110; 1993–2023). High flows are typically driven by late-summer monsoons and averaged 0.67 m^3^/s; (SE = 0.22) in August. Depending on precipitation and irrigation demands, portions of the river may be dewatered [27]. Wildfires are common in the Mimbres River basin, with approximately six fires occurring in the basin since 2013. However, fire severity and proximity to the river greatly influences the impact fires have on habitat of the system. For instance, the Black Fire burned much of the upper Mimbres River basin in 2022 resulting in large-scale ash-flow events throughout July and August (G. Del Piccolo pers. Observation, 22).

The fish assemblage in the Mimbres River is depauperate. Historically, the only native species were Chihuahua Chub, Rio Grande Sucker, and Beautiful Shiner *Cyprinella formosa* [17,28]. The current assemblage consists of Chihuahua Chub and Rio Grande Sucker, alongside nonnative Longfin Dace *Agosia chrysogaster* and Fathead Minnow *Pimephales promelas* [17,19]. Nonnative Rainbow Trout *Oncorhynchus mykiss* and Largemouth Bass *Micropterus salmoides* have also been documented in the Mimbres River, but these species are currently rare due to natural extirpations and management actions [16].

**Fig 1:**
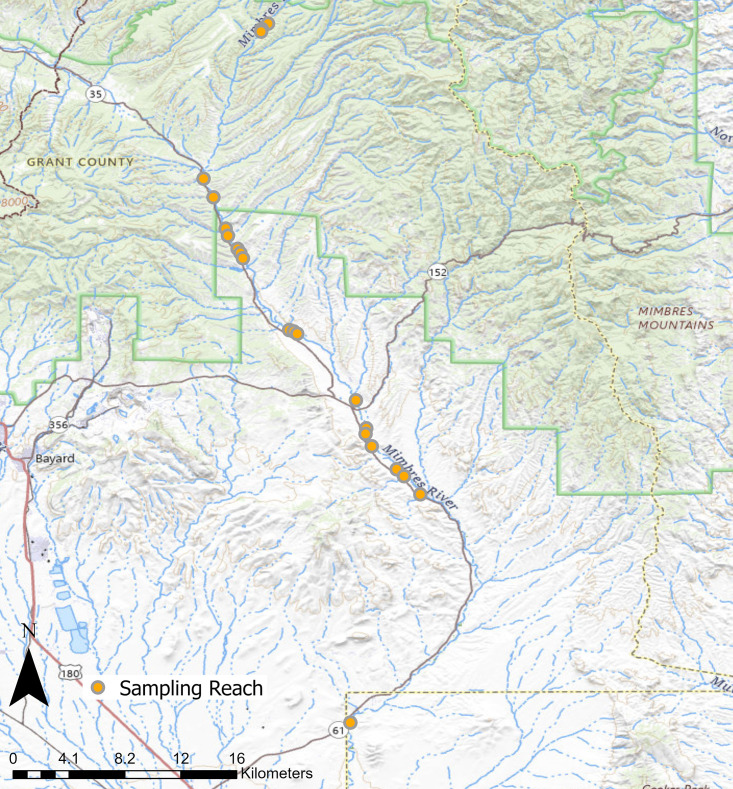
A map of the Mimbres River, New Mexico from USGS National Map Viewer imagery. The locations of sampling reaches (orange circles) is included.

The majority of the Mimbres River is privately owned. As a result, sampling was restricted to properties owned by New Mexico Department of Game and Fish (NMDGF), the Nature Conservancy, the Gila National Forest, and areas with access permission. Within these areas, a total of 21 200-m reaches (approximately 3% of total river) was randomly selected for sampling (Fig 1). Eight of these reaches had previous habitat restoration efforts (e.g., cross vanes, additions of logs and roots wads), while 13 reaches had no previous habitat restoration efforts. Sampling occurred in the winter (December), spring (May), and summer (July–August) from 2021 to 2023. Depending on dewatering events and river access, 13–21 reaches were sampled each season. Each reach was divided into channel units (i.e., pool, riffle, run) for fish and habitat sampling [29,30].

### Fish sampling

The fish assemblage of each channel unit was sampled using a backpack electrofishing unit (LR-24, Smith-Root, Vancouver, Washington) standardized to 3,000 W [31]. All sampling occurred in a single, upstream pass. All available habitat was sampled and fish were collected by two netters. Following sampling, fish were identified, enumerated, and measured (total length; mm). Fish were returned to the channel unit following processing. Each channel unit was sampled three times per season, with one to eight days between resampling events [32,33]. As Chihuahua Chub were the initial focus of this research and high catch-rates of other species limited sampling, Chihuahua Chub were the only species collected during the second and third passes of each season.

### Habitat sampling

The habitat characteristics of each channel unit were measured once per season. The length of each channel unit was measured along the thalweg using a digital rangefinder (Bushnell, Overland Park, KS). Three transects were identified at 25%, 50%, and 75% of the total unit length [29]. Depth, water velocity, and dominant substrate were collected at 10%, 30%, 50%, 70%, and 90% of the wetted width of each transect [29,30]. Depth (0.01 m) was measured using a depth rod. If water depth was less than 1 m, water velocity was measured at 60% of depth [34,35]. If depth exceeded 1 m, water velocity was measured at 20% and 80% of total depth and averaged. Dominant substrate was visually estimated and classified as silt (0.0–0.3 mm), sand (0.3–2.0 mm), gravel (2.0–64.0 mm), cobble (64.0–260.0 mm), boulder (>260.0 mm), or bedrock [30,35,36]. Instream cover (e.g., undercut banks, large wood, root wads) was estimated as a percent of the total area of each channel unit [29,37]. The normalized difference vegetation index (NDVI) was calculated for all 30 m^2^ pixels located within 100 m of the stream channel at each sampling reach using LANDSAT-9 imagery collected during each sampling season (Landsat-9 image U.S. Geological Survey).

### Analysis of Chihuahua Chub habitat associations

Closed, single-season, binomial *N*-mixture models were used to estimate Chihuahua Chub abundance as a function of biotic and abiotic habitat covariates in each channel unit [32]. *N*-mixture models use counts from repeated surveys to estimate abundance (*N*) while accounting for uncertainty in detection probability (*p;* 38) The primary goal of this research was understanding associations between Chihuahua Chub abundance and habitat characteristics. Because we were not specifically interested in estimating abundance, the assumption of demographic closure was relaxed in the analysis [38,39]. Due to evidence of overdispersion in our dataset, abundance was assumed to follow a negative binomial distribution [32,33]. Thus, an overdispersion parameter (*θ*) was included in our models [40]. Models were constructed using a multiple-step approach [32]. To ensure that model coefficients could be compared, continuous variables were scaled to have a mean of 0 and a standard deviation of 1 across each season [41]. An initial set of models using survey-level covariates hypothesized to influence detection probability of Chihuahua Chub was developed. During this stage, abundance was held constant and included a random effect “property” variable [38]. We defined “property” as the parcel of land on which a given reach was located (*n* = 9). This was implemented to account for correlation between channel units subject to similar land-use practices (e.g., grazing) and management (e.g., stocking, habitat restoration). Survey-level covariates hypothesized to influence detection of Chihuahua Chub included electrofishing effort (seconds) and survey time (morning or afternoon). Sampling pass (1^st^, 2^nd^, or 3^rd^) was also included in detection probability models. These models were compared using Akaike Information Criterion adjusted for small sample size (AICc), and the most parsimonious model was used in the second stage of modeling [42].

The relationship between Chihuahua Chub abundance and channel unit covariates was then modeled using *N-*mixture models (Table 1). During this stage, the most plausible detection model was held constant while varying covariates hypothesized to influence abundance (32v). Potential correlation between independent variables was examined using Pearson correlation coefficient [38]. Correlated variables (r ≥ 0.5) were not considered in the same candidate models. All candidate models included a “property” variable as a random effect to account for correlation between similar reaches [38]. Models were compared using AICc and models with a delta AICc of less than 2 were considered plausible [42]. Due to low catch rates (0–4 Chihuahua Chub/season) following the Black Fire in summer 2022, only data from winter 2021 and spring 2022 were used for analysis. This analysis was conducted using the “pcount” function from the “unmarked” package in the program R [43,44].

**Table 1:** Abiotic and biotic habitat characteristics used to evaluate the factors influencing Chihuahua Chub (*Gila nigrescens*) relative abundance in the Mimbres River, New Mexico. Minimum (Min), maximum (Max), mean, and standard deviation (SD) values are provided for winter 2021 and spring 2022.

Variables	Winter 2021				Spring 2022			
	Min	Max	Mean	SD	Min	Max	Mean	SD
Abiotic								
Mean depth (m)	0.08	0.57	0.21	0.10	0.03	0.59	0.16	0.11
Mean velocity (m/s)	0.00	0.65	0.24	0.13	−0.06	0.34	0.08	0.08
Mean wetted width (m)	1.60	9.00	5.10	1.40	0.70	25.00	4.40	3.20
Proportion instream cover	0.00	0.80	0.10	0.16	0.00	0.95	0.11	0.19
Biotic								
NDVI	0.11	0.15	0.14	0.01	0.22	0.43	0.33	0.06
Longfin Dace catch rate (fish/s)	0.00	0.37	0.03	0.06	0.00	0.75	0.04	0.09
Rio Grande Sucker catch rate (fish/s)	0.00	0.09	0.01	0.01	0.00	0.25	0.01	0.02

### Analysis of Rio Grande Sucker habitat associations

Rio Grande Sucker were not repeatedly sampled (this research was initially focused on Chihuahua Chub); therefore, habitat associations of the species were evaluated using generalized linear mixed-effects models across all sampling seasons [41]. Continuous habitat variables were standardized to have a mean of 0 and a standard deviation of 1 across all seasons to aid coefficient comparisons (Table 2; 41). Potential correlations between habitat characteristics were assessed using Pearson correlation coefficient [38]. Correlated variables (r ≥ 0.5) were not included in the same candidate model. A “property” variable was included as a random effect in all candidate models to account for auto-correlation among sampling locations. In addition, a “season” covariate (i.e., winter, spring, summer) was included in candidate models.

**Table 2:** Abiotic and biotic habitat characteristics used to evaluate the factors influencing Rio Grande Sucker (*Pantosteus plebeius*) relative abundance in the Mimbres River, New Mexico. Minimum (Min), maximum (Max), mean, and standard deviation (SD) values are provided for winter, spring, and summer, 2021–2023.

Variables	Min	Max	Mean	SD
**Mean depth (m)**	0.02	0.59	0.21	0.11
**Depth CV**	0.12	2.30	0.47	0.17
**Mean velocity (m/s)**	−0.06	1.10	0.32	0.23
**Proportion cobble**	0.00	1.00	0.49	0.31
**Proportion fines**	0.00	1.00	0.23	0.24
**Proportion instream cover**	0.00	0.95	0.08	0.15
**Mean wetted width (m)**	0.63	29.00	5.60	2.60
**NDVI**	0.06	0.43	0.24	0.10
**Longfin Dace catch rate (fish/s)**	0.00	1.10	0.03	0.09

High catch-rates allowed for habitat associations of Rio Grande Sucker to be investigated separately for subadult (< 100 mm TL) and adult (≥ 100 mm TL) fish [45]. Due to evidence of overdispersion in our data, relative abundance was modeled using a negative-binomial distribution with a log link function [41,46]. Relative abundance was standardized by effort and modeled as a log-transformed offset [47]. Models were compared using AICc and models with a delta AICc of less than 2 were considered plausible [42]. Analysis was conducted using the “glmmTMB” function from the “glmmTMB” package in the program R [44,46].

### Ethics statement

Research was conducted under an approved I.A.C.U.C. protocol, 2213−001 New Mexico State University. Elecrofisher settings were selected to minimize harm to all fish species [31]. Fish identification, enumeration, and measurements were conducted quickly to minimize the amount of time out of the water and all fish were released unharmed.

## Results

A total of 180 Chihuahua Chub was sampled over six sampling seasons, but 96% were sampled during the winter 2021 and spring 2022 sampling seasons (Table 3). The majority (89%) of Chihuahua Chub were sampled in restored reaches, whereas only 43 percent of sampling events occurred in restored reaches (restored reaches were overrepresented in total sampling events as they were less likely to be seasonally dry than non-restored reaches). Chihuahua Chub had a mean total length of 129 mm and varied from 72 to 260 mm. A total of 3,330 Rio Grande Sucker was sampled over all sampling seasons. Although Rio Grande Sucker abundance appeared to decline subsequent to the Black Fire, one year after the fire (summer 2023), the species represented the majority of fish sampled. Rio Grande Sucker averaged 75 mm and varied from 14 to 195 mm. Longfin Dace were the most commonly sampled species (*n* = 5,473) and varied from 10 to 100 mm TL (mean = 61 mm TL). Fathead Minnow and Largemouth Bass were also sampled but were relatively uncommon in the Mimbres River.

**Table 3:** All fish sampled in the Mimbres River, New Mexico, 2021–2023. The number of fish sampled in restored and unrestored reaches and total length information (mean, minimum, maximum) are included.

Species	*n*		Total length (mm)		
	Restored	Unrestored	Mean	Minimum	Maximum
Winter 2021					
**Chihuahua Chub**	54	1	120	72	199
**Rio Grande Sucker**	81	84	93	53	173
**Longfin Dace**	884	11	62	27	96
**Fathead Minnow**	0	11	69	42	83
Spring 2022					
**Chihuahua Chub**	99	19	131	86	260
**Rio Grande Sucker**	130	236	83	22	173
**Longfin Dace**	952	1164	63	19	100
**Fathead Minnow**	0	8	67	53	80
Summer 2022					
**Chihuahua Chub**	4	0	141	132	153
**Rio Grande Sucker**	0	5	99	40	159
**Longfin Dace**	3	3	62	43	82
**Fathead Minnow**	0	5	54	42	67
Winter 2022					
**Chihuahua Chub**	0	1	193	193	193
**Rio Grande Sucker**	0	53	108	14	178
**Longfin Dace**	8	420	64	22	91
**Fathead Minnow**	0	21	58	44	70
**Largemouth Bass**	0	1	120	120	120
Spring 2023					
**Chihuahua Chub**	0	0	–	–	–
**Rio Grande Sucker**	4	38	119	51	192
**Longfin Dace**	14	122	71	24	110
**Fathead Minnow**	0	20	63	55	72
**Largemouth Bass**	0	1	104	104	104
Summer 2023					
**Chihuahua Chub**	1	1	175	172	178
**Rio Grande Sucker**	190	2,450	60	14	195
**Longfin Dace**	530	1,296	55	10	100
**Fathead Minnow**	0	3	67	62	74

Eight *N*-mixture models relating the probability of detecting Chihuahua Chub to survey covariates were considered for winter 2021 and spring 2022. Three models were considered plausible in winter 2021 and one model was considered plausible in spring 2022 (Table 4). In both seasons, the top-ranked detection model included sampling pass and electrofishing effort. Survey time was also included in the top model for spring 2022. The top-ranked detection model for each season was used to model detection in all subsequent models for each season.

**Table 4:** Top *N*-mixture models (∆AICc < 2) describing the relationships between Chihuahua Chub (*Gila nigrescens*) detection (*p*) and variables hypothesized to influence detection of Chihuahua Chub in the Mimbres River, New Mexico. Akaike’s Information Criterion adjusted for small sample size (AICc), delta AICc (∆AICc), the number of parameters (*k*), model weight (*w_i_*), and coefficients are included. Asterisks denote significant variables (*p*-value < 0.05).

Rank	Model	AICc	∆AICc	*k*	*w*_*i*_	Model coefficients					
						Effort	Pass			Survey time	
							1	2	3	Morning	Afternoon
Winter 2021											
1	*p* (pass + effort), *N* (1|property)	237.35	0.00	7	0.42	+0.48*	+0.07*	+0.04	+0.02*	–	–
2	*p* (pass), *N* (1|property)	238.62	1.27	6	0.22	–	+0.10*	+0.06	+0.04*	–	–
3	*p* (pass + survey time + effort), *N* (1|property)	239.27	1.92	8	0.16	–	+0.06*	+0.04	+0.02*	+0.03*	+0.05*
Spring 2022											
1	*p* (pass + survey time+ effort), *N* (1|property)	327.56	0.00	8	0.62	+1.54*	+0.03*	+0.07	+0.05*	+0.06*	+0.03*

**Fig 2:**
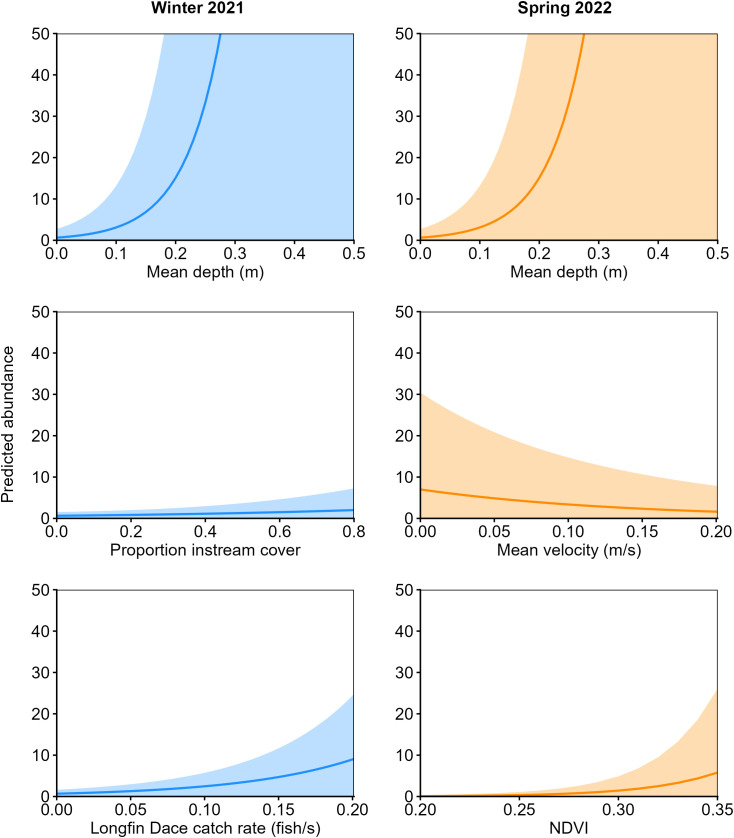
The relationship between predicted Chihuahua Chub (*Gila nigrescens*) abundance (±95% CI) and habitat variables in the Mimbres River, New Mexico, from top-ranked *N*-mixture models in winter 2021 and spring 2022.

Sixteen *N*-mixture models relating Chihuahua Chub abundance to habitat characteristics were considered in the final model set for each season. One model was considered plausible in winter 2021 and two models were plausible in spring 2022 (Table 5). These models indicated that Chihuahua Chub habitat associations were broadly similar between the winter and spring (Fig 2). In both seasons, Chihuahua Chub were positively associated with deep habitats and instream cover. However, differences in habitat associations were evident between seasons. In spring, Chihuahua Chub abundance was positively associated with low water-velocity and NDVI values. The effect of water depth on Chihuahua Chub abundance was greater in spring than in winter. For instance, the predicted abundance of Chihuahua Chub in habitats with a mean depth greater than 0.2 m was 18 times higher in spring 2022 than winter 2021. In the winter, Chihuahua Chub abundance was predicted to be greater than one in habitats with a catch rate of Longfin Dace greater than 0.03 fish/s.

**Table 5:** Top *N*-mixture models (∆AICc < 2) describing the relationships between Chihuahua Chub (*Gila nigrescens*) detection (*p*) and abundance (*N*) and habitat characteristics in the Mimbres River, New Mexico. Akaike’s Information Criterion adjusted for small sample size (AICc), delta AICc (∆AICc), the number of parameters (*k*), model weight (*w_i_*), and coefficients are included. Asterisks denote significant variables (*p*-value < 0.05).

Rank	Model	AICc	∆AICc	*k*	*w*_*i*_	Model coefficients				
						Mean depth	Mean velocity	Instream cover	Longfin Dace	NDVI
Winter 2021										
1	*p* (pass + effort), N [mean depth + instream cover + Longfin Dace + (1|property)], θ	223.36	0.00	10	0.69	+1.05*	–	+0.23	+0.77*	–
Spring 2022										
1	*p* (pass + survey time+ effort), *N* [mean depth + mean velocity + NDVI + (1|property)], θ	310.67	0.00	11	0.35	+1.59*	−0.69	–	–	+1.84*
2	*p* (pass + survey time + effort), *N* [mean depth + instream cover + NDVI + (1|property)], θ	312.28	1.61	11	0.15	+1.91*	–	+0.11	–	+1.70*

**Fig 3:**
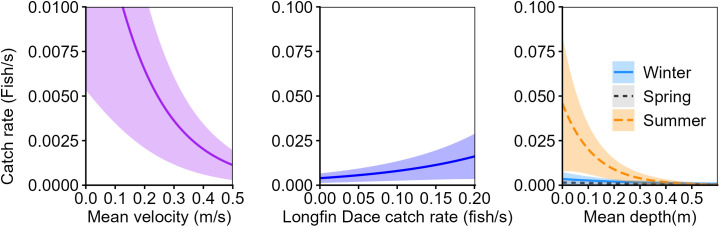
The relationship between predicted catch rate of subadult Rio Grande Sucker (*Pantosteus plebeius*; ± 95% CI) and mean velocity (m/s), Longfin Dace (*Agosia chrysogaster*) catch rate (fish/s), and mean depth by season in the Mimbres River, New Mexico, from top multiple linear regression models (∆AICc < 2) during winter, spring, and summer, 2021–2023.

**Fig 4:**
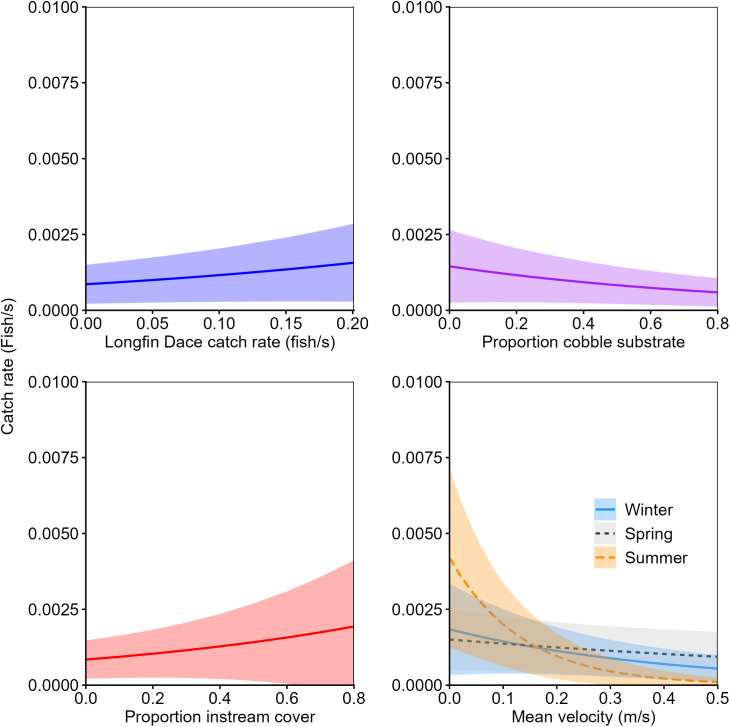
The relationship between predicted catch rate of adult Rio Grande Sucker (*Pantosteus plebeius*; ± 95% CI) and Longfin Dace (*Agosia chrysogaster*) catch rate (fish/s), proportion cobble substrate, proportion instream cover, and mean velocity by season in the Mimbres River, New Mexico, from top multiple linear regression models (∆AICc < 2) during winter, spring, and summer, 2021–2023.

Twenty linear mixed-effects models relating relative abundance to habitat characteristics were considered for subadult and adult Rio Grande Sucker. One model was considered plausible for subadult fish and one model was considered plausible for adult fish. These models indicated that subadult and adult Rio Grande Sucker relative abundance was influenced by similar habitat variables (Table 6). Both age classes of Rio Grande Sucker were positively associated with Longfin Dace relative abundance and low water-velocity (Table 7). For example, catch rates of greater than 0.001 adult Rio Grande Sucker per second were associated with water velocities less than 0.20 m/s during summer and Longfin Dace catch rates greater than 0.054 fish per second. Relative abundance of the species was higher during the winter and summer than the spring. Subadult Rio Grande Sucker were negatively associated with depth, particularly during the summer sampling season (Fig 3). For example, catch rates of greater than 0.001 subadult Rio Grande Sucker per second were associated with habitats with a mean depth less than 0.21 meters during spring low-flows. Adult Rio Grande Sucker were positively associated with instream cover and negatively associated with cobble substrates (Fig 4). During summer, there was a strong negative association between water velocity and adult Rio Grande Sucker relative abundance; whereas, in spring this relationship was weakly positive and in winter it was weakly negative.

**Table 6:** Top linear mixed-effects models (∆AICc < 2) describing the relationships between adult and subadult Rio Grande Sucker (*Pantosteus plebeius*) relative abundance and habitat variables in the Mimbres River, New Mexico. Akaike’s Information Criterion adjusted for small sample size (AICc), delta AICc (∆AICc), the number of parameters (*k*), and model weight (*w_i_*) are included.

Rank	Model formula	AICc	∆AICc	*k*	*w*_*i*_
Subadult					
1	Mean velocity + Longfin Dace + (mean depth × season) + offset(log(effort) + (1|property)	1,582.07	0.00	10	0.95
Adult					
1	Longfin Dace catch rate + proportion cobble substrate + instream cover + (mean velocity × season) + offset(log(effort) + (1|property)	1,043.26	0.00	11	0.90

**Table 7:** Coefficient estimates from the top models (∆AICc < 2) describing the relationship between subadult and adult Rio Grande Sucker (*Pantosteus plebeius*) relative abundance and variables characterizing habitat and season in the Mimbres River, New Mexico. Asterisks denote significant variables (*p*-value < 0.05).

Variable	Estimate	
	Subadult	Adult
**Longfin Dace**	+0.61*	+0.25*
**Mean velocity**	−1.36*	–
**Proportion cobble**	–	−0.34*
**Instream cover**	–	+0.15
**Winter**	+0.66*	−0.11
**Spring**	−7.19*	−6.77*
**Summer**	+2.10*	−0.32
**Mean depth × winter**	−0.09	–
**Mean depth × spring**	−0.19	–
**Mean depth × summer**	−0.67*	–
**Mean velocity × winter**	–	−0.35
**Mean velocity × spring**	–	+0.22
**Mean velocity × summer**	–	−1.51*

## Discussion

Understanding the habitat associations of fish is critical for conserving species that are threatened by habitat loss and/or degradation. The habitat associations we documented for Chihuahua Chub and Rio Grande Sucker can be used to direct future habitat restoration or repatriation efforts for the species, an approach that has been successfully used to conserve rare fish species. For example, Neosho Madtom *Noturus placidus* were positively associated with riffle habitats in the Cottonwood River, Kansas, leading to the construction of artificial riffle habitat [48]. Habitat-use information was used to identify tributaries with suitable biotic and abiotic conditions for the reintroduction of Arctic Grayling *Thymallus arcticus* in the Mantisee River, Michigan [49]. In the Mimbres River, Chihuahua Chub relative abundance was highest in locations where habitat improvements occurred; whereas, Rio Grande Sucker were common throughout the river. For instance, 89% of Chihuahua Chub were sampled in improved habitats; whereas, 14% of Rio Grande Sucker were sampled in restored habitats. Although Chihuahua Chub appeared to benefit from habitat restoration, the species was associated with specific habitats within restored reaches of the Mimbres River. Chihuahua Chub were most often associated with deep, structurally-complex habitats that contained instream cover (e.g., root wads). Rio Grande Sucker were also associated with habitats with instream cover, but tended to occur in low-velocity run habitats. Chihuahua Chub have been reported to prefer deep pools with ample instream cover [17,50]. Rio Grande Sucker have been documented using structurally complex, low-gradient runs in northern portions of the species’ distribution [25,26,51]. Due to the apparent importance of structurally-complex, low-velocity habitats, managers should seek to maintain or restore such habitat to enhance conservation of both species in the Mimbres River.

Habitat heterogeneity can be an important consideration for species conservation due to variability in habitat use by life stage and season [52–54]. Age-0 Candy Darter *Etheostoma osburn* preferred deep, low-velocity habitats; whereas, adults preferred shallow, high-velocity habitats in the New River, West Virginia [55]. Suckermouth Minnow *Phenacobius mirabilis* in the Marais des Cygnes River, Kansas and Missouri, were associated with shallow habitats during the spring spawning season and returned to deeper habitats in early summer [56]. In this study, water velocity and water depth were important determinants of Chihuahua Chub and Rio Grande Sucker relative abundance. However, the importance of water depth and velocity changed by season. Chihuahua Chub were more influenced by water depth in spring compared to winter. Rio Grande Sucker tended to be associated with shallow (subadult) or low-velocity (adult) habitats in the summer but were less associated with these habitats in the winter and spring. Seasonal water variability likely explains the patterns observed for both species. Deep pools may provide refuge for Chihuahua Chub during low-discharge periods typical of the spring. Previous authors noted that Chihuahua Chub were found in deep pools during dry seasons in the Rio Piedras Verdes, Chihuahua, Mexico [50]. Similarly, Rio Grande Sucker may use shallow or low-velocity habitats as refuge from high flows during summer monsoons. As was suggested in previous research [57] this research found that subadult Rio Grande Sucker were associated with shallower habitats relative to their adult conspecifics. Although stage-specific Chihuahua Chub habitat associations were not assessed in this research due to low catch-rates, Miller and Chernoff (1979) noted that they encountered subadult Chihuahua Chub in shallower habitat than adults [50]. Subadult Rio Grande Sucker and Chihuahua Chub may be excluded from deeper habitats due to competition with adult fish or may select different habitats due to predator avoidance or ontogenetic dietary differences [58–60]. Thus, it is uncertain if additional shallow habitats will benefit subadult Rio Grande Sucker and Chihuahua Chub. Nevertheless, observed patterns in seasonal and stage-specific habitat associations suggest that habitat heterogeneity will likely benefit Chihuahua Chub and Rio Grande Sucker conservation.

In addition to habitat, nonnative species are often cited as a major threat to fishes in arid regions [61,62]. Miller and Chernoff (1979) suggested that Longfin Dace negatively influenced Chihuahua Chub via competition [50]. Interestingly, Chihuahua Chub and Rio Grande Sucker were positively associated with the relative abundance of Longfin Dace in the Mimbres River. The diet of Longfin Dace has been reported to overlap with the diets of species that are closely related to Chihuahua Chub (i.e., Headwater Chub *Gila nigra*) and Rio Grande Sucker (i.e., Desert Sucker *Pantosteus clarkii*) in the Gila River, New Mexico [63–66]. Therefore, Longfin Dace have the potential to compete with both species for resources. A reduction of Longfin Dace may increase prey availability for Chihuahua Chub and Rio Grande Sucker and positively affect dynamics rates for the native species (e.g., survival, growth; [67,68,69,70]). However, it is important to note that Longfin Dace do not appear to exclude or reduce the relative abundance of Chihuahua Chub or Rio Grande Sucker in the Mimbres River.

Desert fish populations are often isolated and are at increased risk of extinction due to environmental or demographic stochasticity [71,72]. Dewatering events in 2017 were associated with the extirpation of Carbonera Pupfish *Cyprinodon fontinalis* and Largemouth Shiner *Cyprinella bocagrande* from Ojo Solo Spring, Chihuahua, Mexico [73]. The risk of local extinction can be mitigated by movement between suitable habitats. The local extinction risk of Desert Sucker and Sonora Sucker *Catostomus insignis* was reduced in the Gila River, New Mexico, because the species were able to recolonize unoccupied habitats after local extirpations due to wildfires [74]. Similarly, we found that although catch rates of Rio Grande Sucker declined subsequent to the 2022 Black Fire, the highest Rio Grande Sucker catch rate occurred in summer 2023, one year after the fire, suggesting that the species was resilient to wildfire in the Mimbres River. Chihuahua Chub were less resilient to wildfire and only 4 individuals were sampled across 4 sampling seasons after the fire. Many sucker species have been documented moving large distances [74]. However, there is little information about Chihuahua Chub movement. Although Osborne (2019) documented a single Chihuahua Chub to have moved over 20 rkm, Del Piccolo (2023) reported that the largest movement among 14 fish was 488 m [18,75]. Low discharge coupled with frequent dewatering likely reduced the ability of Chihuahua Chub to move long distances in the system. Increased habitat connectivity may allow Chihuahua Chub and Rio Grande Sucker to recolonize river reaches subsequent to local extirpations [76–78]. Conversely, the difference in response to wildfire between the two species may have been due to differences in the availability of preferred habitats between the two species. Chihuahua Chub were associated with deep habitats which were mostly restricted to restored and undisturbed reaches. Rio Grande Sucker were associated with low-velocity habitats which were relatively common throughout the basin. Therefore, refugia from ash-flows may been more available to Rio Grande Sucker than Chihuahua Chub.

Understanding the habitat associations of sensitive fishes is an important step in planning and implementing conservation efforts [7,8]. This study indicated that Chihuahua Chub are associated with deep, structurally complex pools in reaches that contain abundant riparian vegetation. Rio Grande Sucker were also associated structurally-complex habitat but in low-velocity run habitats. Therefore, habitat restorations should attempt to restore and/or maintain habitats that promote deep pools, low water-velocities, and structural-complexity. Habitat heterogeneity may be important to both species as evidenced by differences in habitat associations between life stages and seasons. Increased availability and connectivity of high-quality habitat may help to improve the resilience of Chihuahua Chub in the Mimbres River. Although restoring habitat quality and connectivity may be challenging due to funding constraints and patchwork landownership, habitat improvements are likely one of the best ways to promote the conservation of Chihuahua Chub and Rio Grande Sucker in the Mimbres River and throughout their distributions.

## Supporting information

S1 File This zip file contains the data and code used in this analysis. (ZIP)
